# Supplementary material for: Mechanisms governing the pioneering and redistribution capabilities of the non-classical pioneer PU.1
Source: Nat Commun. 2020 Jan 21;11:402. doi: 10.1038/s41467-019-13960-2 (PMC6972792; doi:10.1038/s41467-019-13960-2)
Supplement: Supplementary file 7 — Source data [file 41467_2019_13960_MOESM7_ESM.zip › Source_Data/FigureS2/FigureS2B_MotifScanOutput/KmeansCluster12/homerResults/motif34.similar.html]

motif34

## Information for motif34

T
A
C
G
C
G
A
T
A
C
T
G
A
T
C
G
A
G
C
T
G
C
A
T
A
C
T
G
A
C
T
G
C
A
G
T
A
C
T
G
A
T
C
G
G
A
C
T
C
G
A
T
C
A
G
T
  
Reverse Opposite:  

G
T
C
A
C
G
T
A
C
T
G
A
T
A
G
C
T
G
A
C
G
T
C
A
T
G
A
C
T
G
A
C
C
G
T
A
T
C
G
A
T
A
G
C
T
G
A
C
G
C
T
A
A
T
G
C
  

|  |  |
| --- | --- |
| p-value: | 1e-16 |
| log p-value: | -3.787e+01 |
| Information Content per bp: | 1.640 |
| Number of Target Sequences with motif | 85.0 |
| Percentage of Target Sequences with motif | 4.73% |
| Number of Background Sequences with motif | 730.5 |
| Percentage of Background Sequences with motif | 1.59% |
| Average Position of motif in Targets | 91.8 +/- 51.8bp |
| Average Position of motif in Background | 97.3 +/- 58.9bp |
| Strand Bias (log2 ratio + to - strand density) | -0.1 |
| Multiplicity (# of sites on avg that occur together) | 1.06 |
| Motif File: | file (matrix) reverse opposite |

### Similar de novo motifs found

|  |  |  |  |  |  |  |  |
| --- | --- | --- | --- | --- | --- | --- | --- |
| Rank | Match Score | Redundant Motif | P-value | log P-value | % of Targets | % of Background | Motif file |
| 1 | 0.899 | C T A G C G A T A C T G A C T G A G C T C G A T A C T G C T A G A C G T A C T G A C T G A G C T C G A T | 1e-13 | -31.684349 | 0.72% | 0.02% | motif file (matrix) |
